# Identification and functional analysis of secreted effectors from phytoparasitic nematodes

**DOI:** 10.1186/s12866-016-0632-8

**Published:** 2016-03-21

**Authors:** Sajid Rehman, Vijai K. Gupta, Aakash K. Goyal

**Affiliations:** International Center for Agriculture Research in the Dry Areas (ICARDA), Rabat-Instituts-Morocco, P.O.Box 6299, Rabat, Morocco; National University of Ireland Galway, Galway, Ireland

**Keywords:** Nematodes, Phytoparasitic, RNA interference (RNAi), Plant-parasitic nematodes (PPN)

## Abstract

**Background:**

Plant parasitic nematodes develop an intimate and long-term feeding relationship with their host plants. They induce a multi-nucleate feeding site close to the vascular bundle in the roots of their host plant and remain sessile for the rest of their life. Nematode secretions, produced in the oesophageal glands and secreted through a hollow stylet into the host plant cytoplasm, are believed to play key role in pathogenesis. To combat these persistent pathogens, the identity and functional analysis of secreted effectors can serve as a key to devise durable control measures. In this review, we will recapitulate the knowledge over the identification and functional characterization of secreted nematode effector repertoire from phytoparasitic nematodes.

**Research:**

Despite considerable efforts, the identity of genes encoding nematode secreted proteins has long been severely hampered because of their microscopic size, long generation time and obligate biotrophic nature. The methodologies such as bioinformatics, protein structure modeling, *in situ* hybridization microscopy, and protein-protein interaction have been used to identify and to attribute functions to the effectors. In addition, RNA interference (RNAi) has been instrumental to decipher the role of the genes encoding secreted effectors necessary for parasitism and genes attributed to normal development. Recent comparative and functional genomic approaches have accelerated the identification of effectors from phytoparasitic nematodes and offers opportunities to control these pathogens.

**Conclusion:**

Plant parasitic nematodes pose a serious threat to global food security of various economically important crops. There is a wealth of genomic and transcriptomic information available on plant parasitic nematodes and comparative genomics has identified many effectors. Bioengineering crops with dsRNA of phytonematode genes can disrupt the life cycle of parasitic nematodes and therefore holds great promise to develop resistant crops against plant-parasitic nematodes.

## Background

Nematodes are the most abundant multi-cellular animals on earth. Most of the nematodes are simple, colorless and transparent roundworms with relatively little morphological variation. A vast majority of the nematodes is free living, feeding on fungi, bacteria, organic matter, and other nematodes (predators). Only a small percentage of the phylum Nematoda are parasites of animals and plants. Plant-parasitic nematodes (PPNs) have been reported to cause annual crop losses worth $ 173 billion [1]. PPNs are classified according to their feeding and reproduction behaviour. The ectoparasites (e.g., *Trichodorus* and *Xiphinema* spp.) mainly feed on epidermal cells, root hairs or on the outer cortical cells beneath the epidermal cell layer using their stylets. The migratory endo-parasites (e.g. *Aphelenchoides* and *Bursaphelenchus* spp.) penetrate plant tissue through several cell layers and feed on cytoplasm of the cells that they come across. Finally, the sedentary endo-parasites, root-knot nematodes (*Meloidogyne* spp.) and cyst nematodes (*Heterodera,* and *Globodera* spp.) have developed an intimate and long-term feeding relationship with their hosts [2, 3].

To counteract pathogen ingress plants have evolved a two-layered surveillance system which detect either directly or indirectly specific effector molecules from parasites. The first line of defense in plants is established by extracellular immune receptors that recognize pathogen associated molecular patterns (PAMPS) from diverse pathogens. A classical example is the recognition of 22 amino acids in the flagella of bacteria. Recognition by this basal defense system leads to generic defense responses such as cell wall modifications, release of reactive oxygen species, etcetera. But parasites have found ways to breach the basal immunity by suppressing disease signaling with other effectors molecules. These suppressive parasite effectors, however, may induce changes in molecular states of host resistance proteins (Immune receptors) directly or in the host proteins that are being monitored by the immune receptors, so-called R proteins. The probable outcome of pathogen recognition in this second layer of defense is the activation of disease signaling pathways that lead to specific resistances. In many cases effector recognition results in local cell death or a hypersensitive response (HR), thus inhibiting further pathogen infection and colonization. Effector proteins that are being recognized by the products of resistance (R) genes have acquired so-called avirulence (Avr) activity. This gene-for-gene model, which essentially explains the recognition specificity of disease resistance responses in plants, holds true for most biotrophic plant-pathogen interactions [4]. Plant-parasitic nematodes transform host cells into feeding sites, and the most plausible explanation to this transformation is likely to be found within nematode effector molecules.

**Fig. 1:**
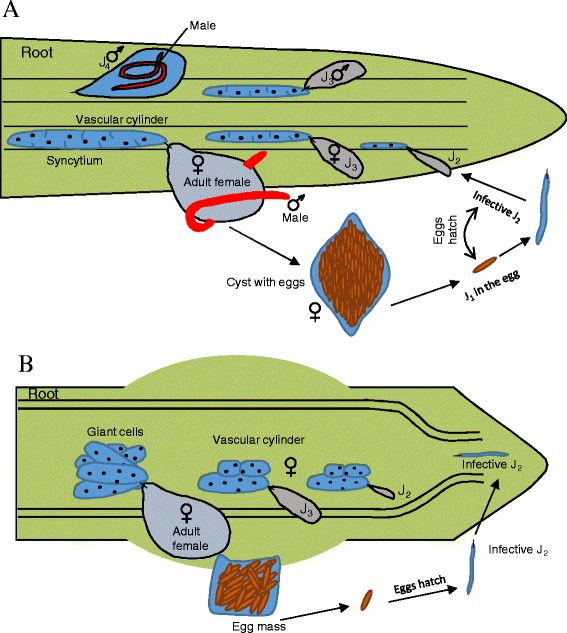
The life cycle of a cyst nematode (**a**) and a root knot nematode (**b**) with different developmental stages

Root knot nematodes (RKNs) and Cyst nematodes (CNs) are obligate plant parasites. Some species of RKNs have a wide host range (*M.arenaria, M. hapla, M. incognita*, and *M. javanica*), however, some species have a restricted host range (*M. partityla*, *M. kralli*, and *M. ichinohei*). Similarly, some of CNs such as *G. rostochiensis* and *G. pallida* have a restricted host range, however, *H. schachtii* has a wide host range (218 plant species). This aspect can aid in their effective control through crop rotation by growing less favorable host plants [5, 6]. The second stage juveniles (J_2_) hatch from the eggs in response to host-plant root exudates and invade the root just behind the apex, preferentially in the differentiation and elongation zone. Plant penetration is achieved by perforating cell walls with the combined effect of physical thrusting of the oral stylet and the enzymatic softening of the cell walls. The infective J2s of RKNs migrate inter-cellularly but CNs nematodes migrate intra-cellularly through the cortex in the direction of the vascular cylinder where they induce specialized feeding structures. RKNs induce giant cells which are formed due to repeated cycles of mitosis without cytokinesis [7]. However, CNs select an inner cortical cell as an initial syncytial cell (ISC) and transforms it into a highly metabolically active cell, which is characterized by small secondary vacuoles, dense cytoplasm, numerous organelles and enlarged amoeboid nucleus [8, 9]. The developing syncytium extends longitudinally along the vascular cylinder by progressive protoplast fusion with neighboring cells through local cell wall dissolution (Fig. 1). Cell wall ingrowths are formed adjacent to xylem elements, facilitating nutrient uptake into the developing syncytium [10, 11]. The giant cells and syncytium act as nutrient sink for several weeks which is continuously replenished by photosynthetic assimilates from the host plant. A high degree of sexual dimorphism has been observed where swollen adults females remain sessile throughout their parasitic life cycle. In contrast, adult males regain motility and become attracted by the females to achieve insemination and fertilization of the eggs. RKN female lays eggs directly on the roots but in case of CNs, the eggs remain inside the body of the gravid female and her remains forms a protective cyst. The first stage juveniles (J1) molt inside the egg and remain dormant for at least 1 year [12].

### Nematode secretions

Nematode secretions are believed to play a key role in the parasitism of plants. These secretions presumably include effector molecules involved in hatching, in self-defense, in movement through plant tissue, and in establishment and maintenance of the feeding structures. Nematode secretions are produced in several different organs, including the cuticle, amphids, the excretory/secretory system, the rectal glands and esophageal gland cells [13]. RKNs and CNs have one dorsal and two subventral esophageal glands. Each gland is a single cell with long cytoplasmic extension that terminates into an ampulla, which serves as a reservoir for secretory granules [14]. As a consequence much of the work done so far has been focused on the products of these esophageal glands. The distinct morphological changes of the esophageal glands at specific stages of parasitism hint towards their differential roles. For example in case of CNs during migration through the plant root, the two subventral glands are large and packed with secretory granules. While shortly after migration ceases, they undergo a strong decrease in cell volume. A larger portion of the genes switched on in the subventral esophageal glands during migration code for cell wall modifying proteins, e.g. ß-1,4-endoglucanases [15], pectate lyases [16] and expansins [17]. In contrast, the dorsal gland shows a remarkable increase in activity during the initiation of the syncytium [18]. Despite advances in molecular biology, still little is known about the host signals that regulate the nematode effector synthesis, packaged into granules and their secretion both in time and space.

### Identification of genes encoding esophageal gland secretions

#### Biochemical analysis

Despite considerable efforts, the identity of genes encoding nematode secretions has long been severely hampered because of their microscopic size, long generation time and obligate biotrophic nature. The direct analysis of the components of nematode secretions is difficult due to the limited amount of material available for analysis. This obstacle was tackled by production of monoclonal antibodies (MAbs) directed against nematode secretions or fractionated homogenate of nematodes. Using MAbs raised against fractionated homogenate of pre-parasitic J2’s of *Globodera rostochiensis*, several nematode β-1-4-endoglucanases were identified [15, 19]. The success rate of the MAb-based cloning approach was rather limited because of many technical disadvantages associated with it. First of all, this technique is more biased towards the effectors being produced at pre-parasitic J2 stage (Cellulases) and many of the effectors being produced rather in minute quantity will not trigger the MAb production and secondly it is quite laborious and time consuming. A significant technical advance was made by the use of chemical compounds such as the neurotransmitter analogue DMT (5-methoxy-N, N-dimethyl tryptamine) to increase pharyngeal pumping and enhanced release of esophageal gland secretions in cyst nematodes [20]. With these compounds, Goverse et al. [20] identified a protein fraction in secretions smaller than 3 kDa showing mitogenic activity on plant protoplasts and T-cell lymphocytes. Similarly Robertson et al. [21] demonstrated in-gel activity of proteases and superoxide-dis-mutase in DMT-induced secretions from *G. rostochiensis*. However, the identity of the genes coding for these activities remains elusive to date. Recently, mass spectrometry was used to identify secreted proteins from pre-parasitic J2’s of *M. incognita* and 486 proteins were identified with functions mapped to protein synthesis and secretion, plant cell wall modification, cell cycle modulation, protection from host defense responses and giant cell formation [22]. Most of these genes were expressed in subventral gland with a rare example of localization of complementary DNA (cDNA) clone in phasmid, an organ not shown to secrete proteins before. Remarkably the secretome of *M.incognita* overlapped with the secretome of the mammalian parasitic nematode (*Brugia malayi*) [23].

### Genomics

#### ESTs

A major leap forward in the identification of parasitism genes was achieved by the work on Expressed Sequence Tags (ESTs). The ESTs are single pass sequences of cDNA clones selected randomly from a cDNA library. In total 116,847 ESTs have been produced from six species of *Meoidogyne* sp., 20,871 from two *Globodera* sp., and 27,256 from two *Heterodera* sp. In addition, about 27,256 ESTs have been deposited in the sequence database from five species of migratory parasitic nematodes (Table 1). Furthermore, NemaGene, NemaBlast, NemaBrowse, NemaSNP and NemaPath are useful tools to annotate nematode derived ESTs/genomic sequences (www.nematode.net). The recently released genomes of *M.incognita* [24], *M.hapla* [25] and *G. pallida* [26] are accelerating the identification and functional annotation of the putative effectors from RKNs and CNs.

**Table 1:** Number of ESTs and genes available in sequence data bases (February 2015)

Sedentary nematodes	# of ESTs	# of genes
*Meloidogyne hapla*	24,452	14420^a^
*Meloidogyne incognita*	63,838	19212^a^
*Meloidogyne chitwoodi*	12,218	
*Meloidogyne javanica*	7,587	
*Meloidogyne arenaria*	5,042	
*Meloidogyne paranaensis*	3,710	
*Globodera rostochiensis*	11,851	
*Globodera pallida*	9,020	16417^a^
*Heterodera glycines*	24,444	
*Heterodera schachtii*	2,812	
Migratory nematodes	# of ESTs	# of genes
*Radopholus similis*	7,382	
*Pratylenchus vulnus*	5,812	
*Pratylenchus penetrans*	1,916	
*Bursaphelenchus mucronatus*	3,193	
*Bursaphelenchus xylophilus*	14,059	

http://www.ncbi.nlm.nih.gov/

http://nematode.net

The letter “a” marks three species with sequenced genome

A set of criteria based on predicted properties of parasitism genes have been used to identify putative nematode effectors. First, selecting proteins with an N-terminal signal peptide for secretion weeds out approximately 90 % of the sequences [27]. The esophageal glands are believed to be important for parasitism, therefore the localization of the transcript within these glands (*in situ* hybridization) is a second important criterion. As a third criterion, the expression of the gene at specific stages of parasitism is being used to further reduce the dataset of potential candidates. Many groups have identified novel parasitism genes such as a pectate lyase [16], a β-1-4-endoglucanase, xylanase [28], a polygalactronase [29], and an ubiquitin extension protein [30] by using this approach. Roze et al. [31] employed bioinformatics approach to explore 12,218 ESTs of *M.chitwoodi*, from three different life stages to identify parasitism genes. After assembling ESTs into 5880 contigs, 398 proteins were predicted to be secreted into the host environment. Furthermore, eight genes were shown to be specifically expressed in esophageal gland cells by *in situ* hybridization [31]. A similar approach was used to identify 50 putatively secreted proteins from *H. glycines* [32].

Owing to the technical difficulties associated with collecting sufficient material from parasitic stages, most of the cDNA libraries have been constructed from pre-parasitic stages. Consequently the current database of ESTs is likely biased towards genes involved in the very early stages of parasitism [33]. In order to clone the genes involved in later stages of parasitism, Gao et al. [34] constructed a pharyngeal gland region specific library by micro-aspirating the contents of the gland cells from parasitic stages of the soybean cyst nematode *Heterodera glycines*. A combination of random sequencing of this gland cell specific library, data mining, and *in situ* hybridization resulted in the identification of 51 novel *H. glycines* esophageal gland-expressed putative parasitism genes.

An even more stringent selection was achieved by combining gland specific micro-aspirated mRNA with subtractive suppressive hybridization (SSH) of messenger RNA (mRNA) from the nematode’s intestinal region. In SSH, the mRNA isolated from intestinal region of nematodes is used as template to produce first strand driver cDNA. The driver cDNA is immobilized on matrix followed by hybridization with another pool of mRNAs isolated from esophageal glands from various parasitic stages by micro-aspiration. Thus cDNAs corresponding to mRNA expressed in both tissues will form a DNA:RNA hybrids, which are removed using a column. Therefore, a unique pool of gland specific mRNAs will be produced. The remaining non-hybridized single stranded mRNA is then used for construction of subtracted cDNA library by reverse transcription polymerase chain reaction (RT-PCR). By using this method, Lambert et al. [35] constructed a cDNA library after differential hybridization of mRNA expressed in posterior and anterior regions of *Meloidogyne javanica* and cloned an esophageal gland specific chorismate mutase (*Mj-cm-1*. Homologues of Mj-cm-1 were found in cyst nematode *H. glycines* [36] and *Globodera pallida* [7].

As discussed earlier, the J2 stage is epiphytic and mobile but the latter stages (J3, J4) are endophytic and remain associated with the same feeding site for the rest of their lives. Thus, it is important to know about the genes expressed during the endophytic stages. SSH was used to identify genes specifically expressed during endophytic J3 stage of *M.incognita* and a glutathione-S-transferase (*GST*) was found to be exclusively expressed and localized to subventral gland of J3 stage. Interestingly, GST protein was not preceded by a classical signal peptide for secretion and hence it can be envisaged that other secretory pathways do exists in parasitic nematodes which are independent of the endoplasmic reticulum-Golgi apparatus. Functional analysis of *GST* by RNA interference showed the importance of this protein in completing life cycle of *M.incognita*. It is hypothesized that GST may safeguard the feeding nematode from the host defense response primarily from reactive oxygen species [37]. There might be more effectors being masked by the presence of unknown secretion signal and which might play crucial role in parasitism. To this end, their functional analysis can be the most probable solution and this exercise is not as high-through put as in other pathosystems.

Most nematode parasitism genes are not expressed constitutively throughout the nematode life, but in a highly coordinated way at specific events in the nematode-plant interactions. Techniques enabling a global analysis of gene expression between different developmental stages allow for the identification of novel parasitism genes up-regulated specifically at the onset of parasitism. Elling et al. [38] used transcript profiling of genes from all stages of *H. glycines* to identify 633 proteins with signal peptide for secretion. Surprisingly, 156 of 633 genes showed strong similarity with proteins from plants and microbes. This finding hints towards possible acquisition of these genes by horizontal gene transfer from other phyla for successful parasitism of host plants [38]. An mRNA finger-printing by complementary DNA- amplified fragment length polymorphism (cDNA-AFLP) allowed a comprehensive analysis of differentially expressed mRNAs isolated from various stages of *G. rostchiensis*. In total 16,500 transcript-derived fragments were analyzed of which 216 were cloned, sequenced, and used for further analysis. The computer program GenEST was used to identify for each of the fragments displayed on gel the matching EST in database [39]. In a recent technical advance, Maier et al. [38] overcome hurdles of getting insufficient gland-cell derived material by elucidating transcriptomes of diverse life stages of various PPNs exclusively from isolated esophageal gland cells. Furthermore, due to differential histochemical staining and morphological differences, dorsal and subventral esophageal gland cells can be separated for further analysis. With this approach, they could extract ~10 – 25 ng of total RNA from 100 dorsal gland cells which could be amplified to get sufficient quantity of RNA to be used subsequently for next generation sequencing platforms. From a single 454 sequencing run, 456,801 reads with an average read length of 409 bp was obtained. In addition to previously identified effectors, numerous novel effectors from *G. rostochiensis, P. penetrans,* and *R. similis* were identified [40]*.*

### Comparative genomics

The fascinating developments in the field of genomics and bioinformatics have allowed scientists to scan the genomes of PPNs to identify their effector repertoires. In an elegant study, Thorpe et al. [41] combined the genome sequence information of *G. pallida* with RNA expression profiles from various developmental stages and identified hundreds of effectors which included 117 novel effectors as well as 128 effector orthologues from other PPNs. Their data is supported nicely by the localization of identified effectors in nematode esophageal glands as well as their localization in different sub-cellular compartments of host plant cell. Despite a comprehensive bioinformatics analysis, 117 effectors of *G. pallida* are novel with no matches in the non-redundant sequence data bases, offering great challenge in future regarding their functional analysis. Even today the estimated number of effector proteins in PPNs is underestimated as many variants of one effector can be produced due to alternative splicing. According to a rough estimate, about 38 % of putative effectors undergo alternative splicing [41].

In *M. incognita,* 90 genes from seven families are involved in cell wall modification [24]. In contra*st, G. pallida* and *M. hapla* have 40 and 41 cell wall degrading enzymes (CWDEs), respectively. Based on the transcriptome profiles, the expression of most of CWDEs was restricted to J2 stage and in males of *G. pallida.* However, the role of CWDEs later in parasitic process cannot be over-ruled as in case of *G. pallida* one arabinogalactan endo 1,4-ß galactosidase was expressed at 7 and at 21 dpi, hinting towards its role in other processes as well. Furthermore, a secreted cellulose binding protein (CBP) from *H. schachtii* interacted with plant pectin-methylesterase which in turn renders degradation of cell wall [42]. The immuno-localization studies have shown that CBP-bearing proteins were being secreted into tomato roots by *M. incognita* and interestingly they were also found to be present in unhatched eggs and close to vulva region. It demonstrates a probable function of these proteins in egg laying process. Hence, it can be envisaged that many effectors from PPNs can have multitude of functions which are not yet known because of our limited understanding and the lack of tools for functional analyses at different parasitic stages during the parasitic process. Remarkably, in *C. elegans* (free living nematode) and *B. malayi* (animal parasite) no CWDEs were reported which shows importance of having such a battery of CWDEs and their importance in whole parasitic process. In addition, their findings also corroborate that there exists no overlap between the effector repertoire of *M. incognita* and *G. pallida* except an overlap in harboring a wide range of CWDEs [26].

### Genome wide scan to identify laterally acquired effectors

Many effectors from PPNs, acquired by lateral gene transfer (LGT) mechanism, have been shown to induce morphological and physiological changes in their hostplants as a part of their parasitism process [43–46]. In case of plant parasitic nematodes, LGT from non-metazoan donors has long been sought to contribute to the enrichment of effector repertoire. With LGT, an organism can gain novel biological functions which renders them to have selective ecological advantages. With the availability of complete genome sequences from a number of PPNs, it is now possible to exploit their genetic information to predict the proportion of their genome being gained by LGT and more importantly to see its role as effectors. By employing comparative genomics on the genome sequences of *M. hapla* and *M. incognita,* Paganini et al. [43] demonstrated that 3.34 % of RKN protein coding genes (680 out of 20,359 protein coding genes) have been acquired as a result of LGT from non-metazoans, predominantly from bacteria and fungi. Some of the bacterial donors include plant pathogens (e.g. *Ralstonia solanacearum, Xanthomonas oryzae, Xanthomonas campestris, Pseudomonas syringae*)*,* symbionts *(e.g. Sinorhizobium meliloti, Methylobacterium nodulans, Mesorhizobium loti*)*,* and rhizosphere dwelling bacteria (e.g. *Burkholderia ambifaria, Agrobacterium radiobacter, Flavobacterium johnsoniae*). Furthermore, many hits were reported from protist (*eukaryotic unicellular organisms*) and fungi [43]*.* Likewise, RKN’s polygalacturonase shows high sequence similarity with GH28 enzymes from *Ralstonia solanacearum*. Furthermore, pectate lyases from RKN and CNs are closely related to pectate lyases from *Clavibacter michiganensis.* Similarly, arabinans and arabinogalactans (family GH43) are more related to their counterparts from bacteria, oomycetes, and fungi. Besides RKNs, it was estimated that 1.25 % of 18,074 protein coding genes from *Bursaphelenchus xylophilus,* the causal agent of pine wilt disease, might have been acquired through LGT from non-metazoans [46]. Strikingly, 146 out of 609 candidate latterly transferred genes have strong sequence identity to genes harbored by bacterial plasmids and hence these mobile genetic entities (plasmids) from bacteria are one of the prime suspects in genome transfer events [43].

It is surprising that the LGT- acquired genes in RKNs did not form a so called “virulence islands” and transposable elements were found to escort them quite frequently. Transposable elements are known to leap through intra- as well as inter-genome and while doing so can transfer genes through a hitchhiking process [47]. In case of *M. incognita*, the acquired genes underwent duplications, forming multi-gene families [45]. It seems that gene duplications started in the common ancestor before the lineage separation into *M. incognita* and *M. hapla*, and in case of *M. incognita* the gene duplication process continued independently. With the emergence of multi-gene families, it can be envisaged that individuals with more copy numbers could have evolutionary success as a result of positive selection pressure. In evolutionary terms the gene duplication can be a sort of adaptive mechanism to cope with new stress/environment, and it can lead to novel gene variants with diversification/specialization of function [48]. The approach adopted by Paganini et al. [43] was quite robust and the authors could confirm that various cell wall degrading/modification enzymes are being acquired due to LGT from their non-metazoan donors. Among them include 12-GH5 cellulases, 3-GH28 ploygalacturonases, and 2-GH43 arabinanases. Furthermore, some new candidates were identified which may have probable function in nematode parasitism such as putative starch-binding CBM20-bearing protein, a mannose 60 isomerase, and a GH25 enzyme. Likewise, Danchin et al. [45] has shown that cellulases, pectate lyases, and expansins are multi-gene families and their relative abundance can be attributed to gene duplication events after their acquisition from respective donors. The acquired genes show over-representation in the functional categories related to metabolism and degradation/modification of carbohydrate polymers (building blocks of plant cell wall). An intriguing finding was the over-representation of proteins involved in protein modification process (protein kinases) and six of the candidate protein kinases have an inherent signal peptide for secretion but experimental evidence is still lacking about their involvement in plant parasitism process. .It can be concluded that LGT events have contributed to genomes and plant parasitic life style of PPNs. Furthermore, a detailed genome search for LGT events in other PPNs can shed more light to assess its evolutionary and biological importance [45].

### Functional characterization of nematode effectors

The list of genes coding for putative parasitism genes from PPN has been growing exponentially over the last two decades. A vast majority of these putative parasitism genes has no match with functionally annotated protein sequences in the non-redundant databases. Earlier it was thought that a fully sequenced genome of *Caenorhabditis elegans*, a free-living bacteriophage, would aid significantly in the functional characterization of putative parasitism genes. However, many genes identified in PPN do not have a functional counterpart in *C. elegans*, thus making its genome sequence a resource with limited value for our understanding of nematode parasitism [34]. Therefore, other more sophisticated methods are being deployed to study the novel parasitism genes that may point at a specific role of the encoded protein in nematode-plant interactions. This section gives an overview of the current methodologies used to study pioneering nematode genes including bioinformatics, protein structure modeling, *in situ* hybridization microscopy, protein-protein interaction studies, and knock-down genes by RNA interference.

### *In silico* analysis of candidate effector proteins

Putative parasitism genes are often first identified as gene fragments in ESTs or transcript derived fragments in cDNA-AFLP that require further efforts such as contig building, sequence cluster analysis, and specific amplification of the cDNA ends to end up with the full gene sequence. Once the full-length sequence is resolved the first important feature to look for in the encoded protein is the presence of N-terminal signal peptide for secretion [27]. Typically, signal peptides are about 24-amino acid long, including N-terminally positioned charged residues, followed by a hydrophobic core, and a more polar carboxy-terminal region [49, 50]. Several computer algorithms build on the SignalP script, such as in PexFinder and SPIT, have been used to distinguish between genes coding for cytoplasmic and secreted proteins of plant pathogens [32, 51]. The next logical step in selecting candidate parasitism genes is therefore to check if the protein includes likely transmembrane regions or retention signals in its sequence. Proteins with an N-terminal signal peptide for secretion but lacking transmembrane regions and other specific retention signal collectively constitute the secretome of the nematode.

Resolving the protein structure may be a key to understand its biological function, and its role in parasitism and/or disease development. Comparative or homology modeling predicts the three dimensional structure of the target protein sequence based primarily on its alignment to one or more proteins of known structure (template). For example, if members of a protein family share >50 % pair-wise amino acid similarity and the structure of one member is determined, it can be used for homology modeling of other family members [52]. Comparative models can be helpful in designing mutants to test the function of proteins [53], to identify active binding sites [54], predicting antigenic epitopes [55], simulating protein-protein docking [56], and confirming a remote structural relationship [57]. Using remote homology modeling Rehman et al. [58] presented a three-dimensional structure model of SPRYSEC-A18. This model was used to construct a consensus structure model for the best matching family members. SPRYSEC is a large gene family comprising of at least 22 members from PCN. Based on modeling study, antigenic peptides were designed on variable loop regions and anti-serum raised was used for immuno-detection of SRPYSEC family members in the PPN secretion [58].

### Localization of candidate effectors in nematodes

The esophageal glands in the plant-parasitic nematode are believed to be an important source for nematode effectors involved in nematode-plant interactions. An important step in the identification of putative effectors is to assay for a specific expression of the candidate effector gene in the esophageal glands by using *in situ* hybridization microscopy on whole mount nematodes. To this purpose the digoxygenin labeled anti-sense cDNA strand derived from a putative parasitism gene can be hybridized with mRNA in target tissue. Following an enzymatic reaction, the hybridization signal can be located, thus allowing determination of spatial expression patterns [59]. Further evidence in support of a role as effector in nematode-plant interactions may be found by using specific antisera for immunolocalization of the corresponding protein in stylet secretions and more importantly in plants infected with nematodes; however, raising specific antisera is not a trivial exercise. The heterologous expression of nematode proteins in bacteria and yeast, which is required for antiserum production, has often proven to be difficult. Nematode proteins have to be genetically fused to hydrophilic carrier proteins, such as maltose binding protein *malE* or glutathione-S-transferase (GST), which reduces the specificity of the antisera. Synthetic peptides designed on the products of candidate parasitism genes have also been used to raise specific antisera to circumvent the difficulties with expressing nematode proteins in bacteria and yeast. The success rate of this approach is low, which makes it not suitable to be implemented in a high-throughput decision scheme. Consequently, in spite of the superiority of the evidence it may provide, *in planta* immunolocalization of candidate nematode effectors has been done for only two nematode genes to date [30, 60].

### Cellular targets of nematode effectors in host cells

The sub-cellular localization of the putative PPNs effectors into the host cell can give us crucial insights into their probable function and this information can be used to short-list the candidate effectors for further functional analysis. Perhaps, the smartest way of parasitizing plants will be to hijack their cellular machinery by inducing transcriptional changes in the nuclei. This notion is correct as many PPNs effectors target host cell nucleus and nucleolus [61–65]. To this end, a fluorescent marker GFP/YFP/RFP is fused with the coding region of putative effector and its localization in host (sub) cellular compartments is monitored. Due to small size of effectors and the truncated expression of the fusion cassette, there can be a passive diffusion of GFP into the nucleus but their localization into nucleolus is considered authentic as nucleolus is refractory to passive diffusion. Using this approach, Tytgat et al. [30] found that an ubiquitin extension protein (*Hs-UBI1*) in the stylet secretions of *Heterodera schachtii* targets the nucleus of host cells. In addition, Gao et al. [34] found that 15 out of 51 candidate effector genes of *H. glycines* include nuclear localization signal suggesting that the host cell nucleus is a major target for nematode effectors. However, despite the absence of a NLS, the C-terminal and N-terminally GFP fused SPRYSEC-19 effector from *G. rostochiensis* localized to the nucleus and nucleolus of tobacco BY2 cells [51]. Although computer predictions could guide us but relying only on them can make our selection of putative effectors for functional analysis from PPNs more biased.

A GFP- fused *M.javanica* effector (*Mj-NULG1a*) localized to the nuclei of the tomato epidermal cell [62]. Similarly, the *in planta* localization of a secreted effector Calreticulin (*Mi-CRT*) was observed by transient expression by Agro-infiltration (ATTA) in tobacco cells. Interestingly, the construct with signal peptide (*Mi-CRT + SP*) was localized in the apoplast, whereas, the construct without signal peptide (*Mi-CRT-SP*) remained in the cytoplasm as predicted by *in silico* analysis. Furthermore, the stable transgenic lines of Arabidopsis expressing a secreted form of *Mi-CRT* were found to be hyper-susceptible to infection by *M.incognita* as well as a fungal root pathogen (*Phytophthora parasitica*). In addition, the susceptibility in nematode effector transgenic plants was linked to the suppression of many defense-related host genes which are normally induced upon treatment with PAMP-molecule elf18 (N-terminal 18 amino acids of Elongation factor Tu; [63]). Recently, the first ever effector targeting the plant peroxisome from *G. pallida* has been reported and peroxiosome is crucial in key metabolic processes such as the production of auxin, jasmonic acid, and the production of hydrogen peroxide. These metabolites play important role in inducing host plant defense response to invading pathogens and it will not be surprising to know that a vast majority of pathogen derived effectors are involved in suppressing host plant defense responses. It can be envisaged that active and passive suppression of plant defenses can be a primary target of secreted nematode effector molecules in the scenario of an intimate parasitic relationship with its host plants.

### Functional analysis of candidate effectors by RNA interference

Without further knowledge of the role of a gene in parasitism a knock-out or knock-down may lead to valuable information on its importance in parasitism. For the majority of the genomic loci of *C. elegans* knock-outs (and knock-downs) have been developed to study the associated phenotype. Complete signal transduction pathway have been resolved by systematically making knock-outs and knock-downs in this nematode species. In 1998, Fire and coworkers discovered a phenomenon in *C. elegans* which is now known as gene-silencing by RNA interference (RNAi). RNAi is the ability of double stranded RNA (dsRNA) to direct sequence specific degradation of homologous RNA. The mechanism of RNAi is thought to be conserved in all eukaryotes. Since its discovery, RNAi has been exploited as a functional genomics tool in insects [66], amphibians [67], and mammals [68].

**Fig. 2:**
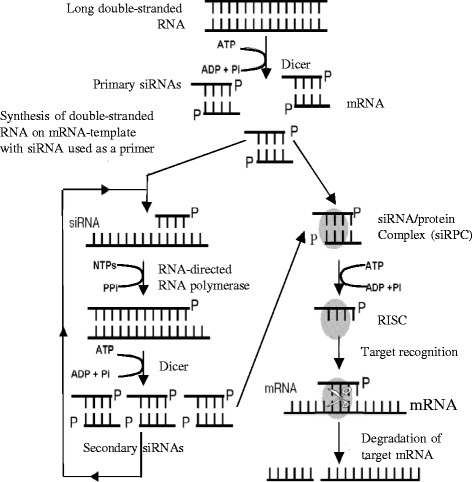
The mechanism of RNAi. Courtesy of V. V. Kuznetsov (2003)

When dsRNA is introduced into a cell it is recognized by a protein named Dicer, an RNase III family nuclease (Fig. 2). Dicer cleaves in an ATP dependent manner the dsRNA into 21–23 bp duplexes of small interfering RNAs (siRNAs) with a 2-nucleotide overhang at 3′ end. These siRNAs are also called primary small interfering RNA’s. siRNAs further associate with an RNA induced silencing complex (RISC) which is activated upon unwinding of the siRNA. The activated RISC, while carrying a single stranded anti-sense strand of the siRNA duplex, scans the whole mRNA population of the cell to find homologous mRNA transcripts. The activated RISC recognizes homologous regions in gene transcripts which results in the cleavage of target mRNA ~12 nucleotides from 3′ end of the hybridized siRNA [69, 70].

The effect of silencing by RNAi is amplified when the primary siRNAs act as primers for synthesis of longer dsRNA using target mRNA as template. This amplification is mediated by RNA-directed RNA polymerase (RdRP). The long dsRNAs is again the substrate of Dicer, resulting in the production of secondary siRNAs, which can lead to target mRNA degradation as well [71]. RNAi functions autonomously in mammalian cells but can be spread systemically to other cells and tissues in nematodes and plants.

In *C. elegans* RNAi occurs when bacteria expressing dsRNA are fed to nematodes, by soaking the nematodes in a dsRNA solution, and by microinjection of dsRNA into the nematodes [72]. Unlike *C. elegans*, PPN have a long generation time, often a sexual mode of reproduction, and an obligate parasitic lifestyle, which have been insurmountable obstacles to achieve knock-outs by genetic transformation. A breakthrough in this field came when Urwin et al. [73] published a method to chemically induce ingestion of exogenous dsRNA in pre-parasitic juveniles. Various chemical compounds (octopamine, resorcinol, serotonin) are known to affect pharyngeal pumping in nematodes which is associated with the release of esophageal gland secretions and, more importantly, the uptake of fluids [73, 74]. Subsequently the protocols for RKNs and CNs were optimized and till now many parasitism genes have been functionally analyzed by RNAi (Tables 2 and 3).

**Table 2:** List of genes silenced by RNAi by soaking method in pant parasitic nematodes

Gene name/genbank accession no.	Putative functions of target genes	Nematode species	Observed Phenotype	References
RNAi by Soaking				
*hgctl*, AF498244	C-type lectin	*H. glycines*	41 % decrease in no. of established nematodes	[73]
*hgcp-I*	Cysteine proteinase	*H. glycines*	40 % decrease in no. of established nematodes	[73]
*gp* ^*c*^ *p-i*	Cysteine proteinase	*G. pallida*	25 % less females ecovered	[73]
*pMiDuoxl*, DQ082753	Dual oxidase	*M. incognita*	Up to 70 % decrease in no. of established nematodes	[84]
			Decrease in fecundity	
*hg-pel*	Pectate lyase	*H. glycines*	Favours male development	[85]
*Gr-eng-l*, AF004523	p-1,4-endoglucanase	*G. rostochiensis*	Reduced no. of established	[75]
*Gr-eng-3*, Gr-eng-4			of established nematodes	[19]
Gr-ams-1, AJ270995	Secreted amphid protein	*G.rostochiensis*	Reduced ability to locate and invade roots	[75]
AY013285	Chitin synthase	*M. artiellia*	Delayed egg hatch	[111]
*Hg-amp-l*, AY883023	Aminopeptidase	*H. glycines*	61 % decrease in number of female reproductive	[112]
*Mi-crt*, AF402771	Calreticulin	*M. incognita*	Not detemined	[74]
*Mi-pg-1*, AY098646	Polygalacturonase	*M. incognita*	Not detemined	[74]
*16D10* (DQ841121-DQ841123)	Secreted peptide	*M. incognita*	74 %–81 %‘ decrease in no. of established nematodes	[89]
*Hg-rps-23*, BF014259	Ribosomal protein	*H. glycines*	Decrease in J2 viability	[113]
*hg-eng-l* , AF006052	p-1,4-endoglucanase	*H. glycines*	Decrease in no. of established nematodes	[85]
*hg-gp*	Function unknown	*H. glycines*	Favours male development	[85]
*hg-cm*	Chorismate mutase	*H. glycines*	Favours male development	[85]
*hg-syv46*, AF273728	Secreted peptide SYV46	*H. glycines*	Decrease in no. of established nematodes	[85]
Mi-gsts-l , EL784458	Glutathione-S transferase	*M. incognita*	52 %–71 % decreased in fecundity	[37]
*Gp-ftp-6*	FMRFamide-like peptides Cysteine proteinase	*G. pallida M. incognita*	Inhibition of motility	[81]
*Mi-cpl-l*			60 % decrease in no. of established nematodes	[114]
*Mi-Cg-l*	Function unknown	*M. incognita*	Avirulence gene being recognized by Mi-1 resistance gene	[87]

**Table 3:** List of genes silenced by *in planta* RNAi method in pant parasitic nematodes

Gene name/genbank accession no.	Putative functions of target genes	Nematode species	Observed Phenotype	References
*In planta* RNAi				
AW871671	Integrase	*M. incognita*	>90 % reduction in established nematodes	[96]
AW828516	Splicing factor	*M. incognita*	>90 % reduction in established nematodes	[96]
*16D10* (DQ841121-DQ841123)	Secreted peptide	*M. arenaria,*	63 %-90 % reduction no. of galls and gall size	[89]
		*M. incognita,*		[115]
		*M. javanica, M. hapla,*		
		*M. arenaria*		
*MSP*	Major sperm protein	*H. glycines*	Up to 68 % reduction in nematode eggs	[95]
*MjTIS-11*	Putative transcription factor	*M. javanica*		[97]
*Hg-rps-3a*, CB379877	Ribosomal protein 3a	*H. glycines*	87 % reduction in female cysts	[98]
*Hg-rps-4*, CB278739	Ribosomal protein 4	*H. glycines*	81 % reduction in female cysts	[98]
*Hg-spk-1*, BI451523.1	Spliceosomal SR protein	*H. glycines*	88 % reduction in female cysts	[98]
*Hg-snb-1*, BF014436	Synaptobrevin	*H. glycines*	93 % reduction in female cysts	[98]
*4G06*, AF469060	Ubiquitin-like	*H. schachtii*	23 %-64 % reduction in developing females	[94]
*3B05*, AF469058	Cellulose binding protein	*H. schachtii*	12 %-47 % reduction in developing females	[94]
*8H07*, AF500024	SKP1-like	*H. schachtii*	>50 % reduction in developing females	[94]
*10A06*, AF502391	Zinc finger protein	*H. schachtii*	42 % reduction in developing females	[94]
*Y25*, CB824330	Beta subunit of the COPI complex	*H. glycines*	81 % reduction in nematode eggs	[99, 116]
*Prp-17*, AF113915	Pre-mRNA splicing factor	*H. glycines*	79 % reduction in nematode eggs	[99]
*Mispc3*, Miduox		*M. incognita*	Reduction of nematode number root, retarded female development	[117]
*Cpn-1* , GU074018	Unknown protein	*H. glycines*	95 % reduction in nematode eggs	[99]
Tyrosine Phosphatase, Mitochondrial stress-70 protein precursors, Lactate dehydrogenase		*M. incognita*	Reduced no. of established females	[100]
*Mi-Rpn7*		*M. incognita*	Reduction in reproduction and motility	[118]
Parasitism gene 8D05		*M. incognita*	Reduction in gall number	[110]
Calreticulin-*Mi-CRTN*		*M. incognita*	Reduction in gall number	[119]
Fatty acid and retinol binding protein *(Mj-far-1 )*		*M. javanica*	Ceased development of nematodes along with reduction in giant cell number	[102]
FMRFamide-likepeptides (*flp-14,flp-18*)		*M. incognita*	Reduction in gall number, fecundity, female development and increased root growth of transgenics	[101]
*Pv010*		*P. vulnus*	Reduced nematode multiplication with no visible lesions	[120]
Effector gene, *Mc16D10L*		*M. chitwoodi*	Reduction in fecundity and pathogenicty	[90]
Effector gene, *Gp-hyp*		*G. pallida*	Reduction in nematode parasitism	[121]

### RNAi by soaking

The preparasitic juveniles of PPN are incubated in the concentrated solution of dsRNA and the ingestion is induced by species specific neurotransmitters and it triggers the transient silencing of an endogenous target gene [73]. With the availability of genome sequences and expression data, the number of putative parasitism genes being identified from phytoparasitic nematode is constantly increasing. For few effectors, a biological function can be predicted with high probability due to sequence homology with annotated sequences from the database; for example, secreted cell wall degrading enzymes. Transient knocking down of PCN cellulases lead to reduced infectivity which could be explained by reduced root penetration [19, 75]. However, most of the effectors from RKNs and CNs are pioneers with no sequence similarity and attributing functions to such a large number of putative effectors is offering lot of challenges in terms of high throughput functional analysis screens [76]. So far, in vitro RNAi has been successful in knocking down of 40 phytonematode secreted effectors from five different genera with most of the success stories reported from RKNs and CNs. However, parasitism genes from other migratory endoparasitic nematodes such as *Radopholus similis* [77], and *Bursaphelenchus xylophilus* [78] have also been successfully silenced.

Despite success stories, many laboratories have reported that the soaking method seems to work for certain genes, while other putative parasitism genes seem refractory to RNAi by this method. In brief, factors that influence RNAi in plant parasites are the length of target dsRNA fragment, topology of the fragment, incubation time in dsRNA solution, durability of silencing and the target tissue in the nematode. At least in *G. rostochiensis* for *Gr-eng-3/eng-4*, it was found that dsRNA designed on either the 5′ or 3′-end of the target sequence did not make a significant difference. In addition, long dsRNA molecules (~600 bp) were more effective than shorter fragments (150 and 300 bp; Rehman et al., [79]. However, in the gastrointestinal parasitic nematode *Trichostrongylus colubriformis* a 22 bp siRNA was shown to be far more efficient than the longer dsRNA in inducing RNAi [80]. FMRF amide-like peptides from *G.pallida* (*Gp-ftp-6*) were silenced efficiently by dsRNA of 316 and 227 bp from 3′ end, respectively. However, 88 bp region from 5 ′end did not produce silencing phenotypes [81]. This study demonstrates that the selection of sequence is more important than the length of the silencing fragment. Furthermore in case of *M.incognita*, both the full length transcript (271 bp) as well as the coding sequence (42 bp) of *16D10* were found equally potent in reducing the transcript level by >90 % [82]. Recently, small interfering RNA of 21 bp was shown to be effective in silencing of FMRFamide-like peptides in *G.rostochiensis* and *M.incognita* [83]. This is the first report describing a direct application of siRNA to plant-parasitic nematodes but it remains to be determined if this could be equally efficient strategy in silencing genes from esophageal gland cells. Apparently, the use of siRNAs can bring more target specificity but the secondary RNai molecules generated due to amplification by RdRp should not be neglected as well.

The soaking duration in dsRNA solution seems to be a crucial factor in determining the silencing efficiency of the target gene in phytonematodes. The first report of RNAi in plant-parasitic nematodes suggested that soaking in dsRNA for 4 h would be sufficient to achieve RNAi of genes in the potato cyst nematode *G. pallida* [73]. However, it was found out that an incubation time of at least 24 h was of particular importance to achieve RNAi of endoglucanase (*Gr-Eng 1*) in *G. rostostochiensis* [75]. Furthermore, longer incubation in the highly concentrated dsRNA of Gr-eng-3 (~40 h) was more potent than 24 h soaking. In addition, a noticeable knock-down of SRPYSEC-19 could be observed only after at least 40 h soaking in dsRNA solution. Depletion of *flp-12* transcript in *G.pallida* was observed after incubation time of 18–24 h and pre-parasitic J2’s were unable to migrate in the host plant roots. Strikingly for other *flp* genes, an incubation period of 2–7 days was necessary to observe extreme phenotype [81]. In *Meloidogyne* spp., incubation of J2’s for 4 h results in target transcript level reduction accompanied by strong phenotype as well [74, 82, 84]. Interestingly, the transcript level of glutathione-S-transferase (*Mi-gsts-1*) gene was reduced significantly even after incubation period of 1 h in *Mi* [37]. It seems, therefore, that the species of the nematode, and the gene which is targeted by the dsRNA, may both determine the minimal incubation time required to achieve RNAi.

The persistence of the RNAi effect in plant-parasitic nematode also seems to be quite variable. Rosso et al. [74] soaked the J2’s of RKN (*M. incognita*) in dsRNA of calreticulin (*Mi-crt*) and a polyglactronase (*Mi-pg-1*) and found out that the knock-down was optimal after 20 and 44 h of soaking, respectively. But, for both genes the transcripts regained their normal levels after 68 h of treatment. Furthermore, the transcript level of *Mi-gsts-1* remained effective for 28 h post-incubation and regained normal transcript level after 48 h [37]. In contrast, Urwin et al. [73] showed reduced transcript levels of major sperm protein *Gp-msp* for 14 days post treatment. And, Bakhetia et al. [85] showed that the levels of a cellulase mRNA were back at normal levels beyond 10 days post treatment with dsRNA. It remains to be determined if RNAi phenotype can be inherited to next generation as is the case of free living nematode, *C. elegans*, where the phenotype remained effective over 80 generations [86]. So far there has been one report showing the silencing phenotype to be inherited for five generations of *M. javanica* after the parental J2’s were exposed to dsRNA of *Cg-1* which encodes a avirulence gene [87]. Further investigation are needed to see if the various longevities observed for RNAi are correlated with the tissue in which the target gene is expressed, and the transcript turnover rate of the target gene.

A further complicating factor in these studies is the storage capacity for proteins in the nematodes. Esophageal gland secretions are expressed and stored in secretory granules in the gland cells well ahead of the anticipated time of their deployment by the nematode. In spite of a profound effect on the transcript level in dsRNA treated nematodes, this may not translate in anticipated phenotype as reduced transcript level does not correlate with reduced protein level. It was observed that despite a significant reduction in cellulase transcripts in dsRNA-treated nematodes, protein levels remained unchanged (Rehman et al., unpublished data). Likewise, Rosso et al. [74] have made similar observations for the *Mi-pg-1* gene in *M. incognita*. It has also been observed that even though the transcript level of *Mi-gsts-1* was reduced by 90 % after RNAi treatment, the GST enzyme was detectable until 24 h post treatment [37]. If, therefore, the storage capacity for secretory proteins last long enough such that it approaches the time when the mRNA expression recovers from the dsRNA treatment then the actual window for RNAi to achieve a phenotype may be small.

An extreme care should be taken while drawing inferences about the observable phenotypes in phtonematodes followed by RNAi treatment of a particular gene of interest. Direct physiological observations are difficult because the post RNAi stages of nematodes are inside the roots unless an observable phenotype has been inferred due to sequence homology to a well annotated gene. For example, silencing of cell wall degrading enzymes should result in reduced infectivity which is correlated with reduced penetration [19]. Another example is of *flp* genes which encode neurotransmitter and their transient silencing should result in lack of migration ability of nematodes [83]. When scoring for phenotype of target genes with no homology in the sequence database, classically the involvement of a gene is parasitism will be attributed to reduced number of feeding sites established, leading to reduced number of progeny or more male to female ratio. If the target gene is necessary for development, for sure RNAi of that gene will lead to developmental arrest and care should be taken to attribute the developmental failures to genes required for parasitism [88]. If functional redundancy is present then silencing of one member of the entire gene family will lead to subtle phenotypes which could easily be overlooked.

It can be concluded that RNAi by soaking in dsRNA is a valuable tool for studying nematode genes that are suspected to be involved in parasitism. However, because of the transitory nature of the RNAi following dsRNA by soaking in these nematodes, its use should be limited to the early stages of parasitism. To study genes throughout the parasitic cycle of the nematode, including later parasitic stages, a continuous exposure to dsRNA to nematodes is more appropriate. In the next section, we will discuss a second approach to achieve RNAi in plant-parasitic nematodes by a continuous exposure to host-generated dsRNA.

### Gene knock-down by host generated dsRNA (HIGS)

A short exposure to dsRNA seems to induce a transitory RNAi in plant-parasitic nematodes. This phenomenon makes the RNAi by soaking pre-parasitic juveniles in dsRNA of limited value for genes with constitutive expression and for genes expressed later in the parasitic cycle. In order to achieve a constant delivery of dsRNA to the feeding nematode, host plants could be engineered to express dsRNA molecules of a target gene from PPN. The parasitic nematodes can in principle ingest dsRNA molecules directly or siRNAi molecules derived from preprocessing of long RNAi molecules by host RNAi machinery. The phenomenon by which an exogenous dsRNA is being expressed *in planta* and the uptake of siRNA by the pathogen results in the endogenous gene silencing is referred as host induced gene silencing (HIGS). The advantage of this approach is even if target mRNA is not expressed in the pre-invasive J2 stage, constitutive expression and synthesis of dsRNA/siRNAs in the cytoplasm of these transgenic plant cells may ensure depletion of target transcripts in later stages as nematode will remain associated with the same feeding site for his entire life cycle and the ingestion of dsRNA/siRNAs will lead to silencing of the endogenous nematode gene.

Since 2006, different research groups have reported reduced infectivity of nematodes by expressing dsRNA in host plants. Huang et al. [89] showed that transgenic *Arabidopsis thaliana* plants expressing dsRNA to the *M. incognita* gene *16D10* resulted in 69–92 % reduction in egg count with an overall suppression of nematode development by 74–81 % as compared to control untransformed plants. The *16D10* gene encodes a conserved secretory peptide in four root knot nematode species (*M. incognita*, *M. arenaria, M. javanica,* and *M. hapla*). Overexpression of this peptide in plants stimulates root growth and molecular analysis suggests that it acts as a ligand for a SCARECROW-like transcription factor of host plant [82]. The authors also showed the presence of siRNAs in transgenic plants, and a significant correlation was observed between levels of siRNAs and nematode resistance. More recently, potato cultivars expressing dsRNA of an effector from *M.chitwoodi, Mc16D10L* (Orthologue of *M.incognita 16D10*), showed resistance phenotype to *M.chitwoodi*. In addition, the RNAi effect was inherited in future generations of *M.chitwoodi* as well [90, 91]. Furthermore, *in planta* expressed dsRNA of a secreted effector from *H.schachtii* (*Hs4F01*) showed reduced transcript as well as infection level. As *HsF01* is 33 % identical at amino acid level to Arabidopsis annexin-1, it is speculated that *HsF01* may disrupt cellular metabolism in favor of nematode development by mimicking plant annexin function [92]. Likewise, transgenic Arabidopsis expressing dsRNA of a putative effector (*Hssyv46*) from *H. schachtii* showed reduced number of females being developed as the target gene was silenced in the dorsal gland [93]. Furthermore, targeting four parasitism genes from *H.schachtii* by host delivered dsRNA in Arabidopsis rendered them resistant [94]. It is suggested to co-express nematode gene as well as dsRNA in the same plant to increase the population of siRNA due to amplification step in plants [33].

Nematode developmental genes program the entire life cycle of nematodes ranging from embryogenesis, transformation through larval stages (J1-J4), and reproduction. Functional genomics of *C.elegans* has put more confidence in selecting essential genes required for the biology of nematode and almost all of the selected candidate genes from parasitic nematodes with lethal phenotype have high sequence homology with *C.elegans* genes. Transgenic soyabean plants expressing dsRNA of a major sperm protein compromised the reproductive potential of *H.glycines* [95]. However, Yadav et al. [96] followed a somewhat different approach and demonstrated that transgenic tobacco lines expressing dsRNA to housekeeping genes of *M. incognita* (Integrase and splicing factor) provided effective resistance against RKNs. Remarkably, nematodes recovered from these transgenic plants exhibited a knock-down of mRNA’s of both integrase and splicing factor, which were targeted in this experiment. Similarly, nematodes feeding on transgenic tobacco expressing dsRNA of *MjTis11*, a zinc finger type transcription factor expressed in eggs and eggs producing females, showed depletion of target transcript in these stages although it did not result in a significant decrease in fecundity or egg hatching rate [97]. In addition, *in planta* delivery of dsRNA of four different genes necessary for mRNA metabolism from *H.glycines* resulted in reduced number of cysts recovered showing their importance as potential disease resistance targets [98, 99]. Similarly, 92 % reduction in gall formation was observed on transgenic soybean roots expressing RNAi construct of tyrosine phosphatase gene of *M.incognita* [100]. In another study, silencing of two neuropeptides of *M.incognita* (*flp-14*, and *flp-18*) in tobacco transgenic plants affected badly the infectivity as well as reproduction potential of nematodes [101]. Likewise, impaired female development as well as reduction of giants cell numbers was observed after the J2’s of *M.javanica* infected the transgenic tomato expressing a hairpin construct of gene encoding fatty acid and retinol binding protein [102].

Most of the reports of successful application of host-delivered dsRNA to achieve RNAi in PPN involved root-knot nematodes. While many laboratories working with cyst nematodes have failed to achieve similar outcomes for these parasites. It is possible that elements in the biology of the cyst nematodes preclude uptake of dsRNA or siRNA from host plants. For instance, root-knot nematodes and cyst nematode are different in size exclusion limit of stylet orifice. It has been observed that cyst nematodes like *G. pallida* and *H. schachtii* do not ingest dsRNA efficiently, while *M. incognita* readily took up the dsRNA molecules [84]. It is not clear if the RNAi by host-delivered dsRNA is conditioned by the uptake of dsRNA molecules or by the uptake of plant-generated siRNA. Root-knot nematodes and cyst nematodes may differ in the susceptibility to siRNA or may differ in their endogenous processing ability of dsRNA. Alternatively, the promoters that have been used to control dsRNA expression may be regulated differently in feeding sites of root-knot nematodes and cyst-nematodes. These issues, along with many more that could be speculated on, underline the need to prioritize further investigation on the RNAi pathways in root-knot nematodes and cyst nematodes.

### Heterologous expression of parasitism genes in plants

Eliminating one specific nematode gene from the molecular interplay of host and parasite as described above is likely to provide insight into the importance, if not the role, of that particular gene. Conversely, constitutive overexpression of a nematode gene in a host plant followed by nematode infections may also shed light on the role of that particular gene in the interaction. Nematode effectors induce major morphological and physiological changes in a host plant such that it sustains nematode feeding for a long time. The phenotypic changes induced by the over-expression of nematode parasitism genes may result in a direct effect on plant growth and development that can be related to the nematode-induced changes in a host plant. To date, the best characterized example of a profound effect of a nematode gene on plant morphology is the over expression of a nematode chorismate mutase from *M. javanica* (*MjCM-1*). CM is secreted after 3 dpi into the host cell cytoplasm and transgenic expression of *MjCM-1* in hairy roots of soybean resulted in the suppression of auxin (IAA) synthesis, reduced vascularization and lack of lateral root development. This observed phenotype could be rescued upon exogenous application of auxin. These findings suggest an important role of CM in the early stages of giant cell formation [60]. One secreted effector (*Hg-SYV46*) of *H. glycines* shares a motif with CLAVATA3/ESR-related (CLE) protein family of Arabidopsis. The ectopic expression of *HG-SYV46* not only rescued the *clv3* mutant of Arabidopsis but its overexpression in wild type plants produced a restricted root phenotype which is a typical phenotype observed after over-expression of *CLE* family members of Arabidopsis [103]. Strikingly, the constitutive expression of a dorsal gland protein from *M.incognita* (*Mi-7E12*) rendered the tobacco plants susceptible with significantly higher numbers of gall formation than control un-transformed tobacco plants. Furthermore, the giant cell morphology and physiology showed a typical example of compatible interaction with increased number of vacuoles and cell wall invaginations. This data clearly demonstrates the role of secreted nematode effector in promoting compatible interaction with its host plants [104]. Similar results were reported for *M.javanica* effector (*Mj-NULG1a*) where *in planta* RNAi resulted in attenuation of parasitic ability and the ectopic expression rendered Arabidopsis plants susceptible to nematode infection [62].

### Molecular targets of nematode effectors in host cells

Nematode effectors are likely to interact with host plant molecules in and outside the host cells. The identification of molecular targets of nematode secreted effectors into the host plant cytoplasm can unfold their parasitism success. Yeast two hybrid (YTH) has been used extensively in a wide range of organisms and its use in nematode research is of no exception. A secreted peptide 16D10 from *M. incognita* was shown to interact with two SCARECROW-like transcription factors from tomato root library. This small peptide has been shown to be conserved in four RKN species and its homologue is absent in CNs. As *16D10* modulate root growth and differentiation, it can be hypothesized that it can re-program root cell proliferation [82]. Similarly, a secreted protein from *H. schachtii* interacted with spermidine synthase 2 (*SPDS2*) from *A. thaliana* in YTH. Further analysis revealed that the expression level of SPDS2 is elevated upon nematode infection and plants with higher expression of SPDS2 renders plants susceptible to *H. schachtii* [105]. Likewise, SPRYSECs constitute a large family of secreted proteins from *G. rostochiensis*, consisting only of a B30.2/SPRY domain and a signal peptide for secretion. It was shown that a nematode secreted protein (effector SPRYSEC-19) physically associated with the C-terminal part of the leucine rich repeat domain of a CC-NB-LRR protein (SW5F) but did not lead to activation of host defense response. Hence, it was speculated that SPRYSEC-19 and SW5F can be evolutionary intermediates approaching on or departing from a classical gene-for-gene relationship. Alternately, binding of SPRYSEC-19 to SW5F leads to suppression rather than activation of disease-resistance pathways. Indeed, the authors latter showed that SPRYSEC-19 suppressed the programmed cell death and disease reaction mediated by different resistance genes [106]. Furthermore, the genome annotation of *G. pallida* revealed the presence of 180 genes sharing high similarity with SPRY-domain containing proteins from *G. rostochiensis* [41] which further highlights its importance in whole parasitic process*.* Other host proteins that have been shown to interact with nematode secreted effectors include a pectinmethylesterase [42], an auxin influx transporter (LAX3; [107]), a β-1,3-endoglucanase [108], a papain-like cysteine protease (Rcr3^pim^; [109]) and an aquaporin tonoplastic intrinsic protein [110].

The bait-prey interaction in YTH reconstitutes the GAL4 transcription factor in the nucleoplasm of yeast cells. While some of the parasitism gene products may target the nucleus of host cells, others may interact with host proteins in other subcellular compartments of host cells. Physical interactions that are found in the nucleoplasm of yeast in YTH may not occur in the cytoplasm of host cells. Therefore, in order to assess the biological relevance of physical interactions found in Y2H they need to be confirmed independently by other methods such as co-immuno-precipitation and pull down assays in vitro or preferably in plant cells.

## Conclusion

Plant parasitic nematodes pose a serious threat to food security of various economically important crops. The use of host plant resistance by traditional breeding to combat the infection of PPN is not very effective due to lack of novel sources of resistance on one hand and on the other hand the race-specific resistance even if found, can easily be overcome due to the emergence of more virulent biotypes. Restricting yield loses due to the use of nematicides has deleterious effect on the environment and most of them have been banned from developed countries. Due to the limitations of the existing control measures, it is of utmost importance to develop new control strategies. There is a wealth of genomic and transcriptomic information available on plant parasitic nematodes and comparative genomics had identified many parasitism genes. The next challenge is how to relate those candidate genes to nematode parasitism by functional analysis. Despite disadvantages, RNAi has revolutionized the functional genomics in parasitic nematodes. The plant parasitic nematode genes targeted so far by RNAi can be divided into three classes based on the annotation of the target gene like 1) Putative parasitism genes, 2) Genes required for nematode development, and 3) Housekeeping genes. In principle, host-delivered RNA interference (HIGS) triggered silencing of genes in plant-parasitic nematodes may prove to be a novel disease resistance strategy with wide biotechnological applications. Bioengineering crops with dsRNA of phytonematode genes can disrupt the developmental life cycle of parasitic nematodes and therefore holds great promise to develop resistant crops against plant-parasitic nematodes. It can be advocated that the introduced RNAi hairpin in host plants is not translated into a protein and it has great target specificity as only the root parasites will be affected only. Target specificity can even be fine-tuned further by excluding homologous sequences in host plants to avoid any off-target effects, use of nematode inducible promoter in the roots, and the selection of nematode species specific parasitism genes. The new emerging genomic technologies hold great promise in enhancing our understanding of nematode infection process and using this knowledge in turn to engineer crops for a sustainable yield potential.

## Abbreviations

AFLP: amplified fragment length polymorphism
Avr: avirulence
cDNA: complementary DNA
CNs: cyst nematodes
CWDEs: cell wall degrading enzymes
CLE: CLAVATA3/ESR-related
dsRNA: double stranded RNA
EST: expressed sequence tag
GST: glutathione-S-transferase
GFP: green fluorescent protein
HIGS: host-delivered RNA interference
HR: hypersensitive response
ISC: initial syncytial cell
J: juveniles
LGT: lateral gene transfer
mRNA: messenger RNA
MAbs: monoclonal antibodies
PAMPS: pathogen associated molecular patterns
PPNs: plant-parasitic nematodes
RFP: red fluorescence protein
RT-PCR: reverse transcription polymerase chain reaction
RNAi: RNA interference
RISC: RNA induced silencing complex
RdRp: RNA-directed RNA polymerase
RKNs: root knot nematodes
siRNAs: small interfering RNAs
SPDS2: spermidine synthase 2
SSH: subtractive suppressive hybridization
Hs-UBI1: ubiquitin extension protein
YTH: yeast two hybrid
YFP: yellow fluorescence protein
